# Association between positive control in self-perceptions of aging and motoric cognitive risk syndrome among Chinese community-dwelling older adults: a cross-sectional study

**DOI:** 10.1186/s12877-023-03934-x

**Published:** 2023-04-03

**Authors:** Guiying Yao, Yanyan Luo, Huimin Wu, Min Gao, Junjun Sun

**Affiliations:** 1 School of Nursing, Xinxiang Medical University, 601 Jinsui Road, Xinxiang, 453003 Henan China; 2 Xinxiang Key Laboratory for Chronic Disease Basic Research and Intelligent Care, Xinxiang, Henan 453003 People’s Republic of China; 3 School of Nursing, SIAS University, Xinzheng, Henan 451150 People’s Republic of China

**Keywords:** Positive control, Self-perceptions of aging, Motoric cognitive risk syndrome, Community-dwelling, Older adults

## Abstract

**Background:**

Self-perceptions of aging (SPA) are important psychosocial factors that lead to a wide range of outcomes including dementia. However, the relationships between positive SPA and motoric cognitive risk syndrome (MCR) which is a predementia syndrome are still unknown. This study aimed to reveal the associations of positive control and aging awareness of SPA with the risk of MCR and its components.

**Methods:**

A cross-sectional design was conducted among 1137 Chinese community-dwelling older adults. Positive control and aging awareness were defined by two dimensions of SPA (Positive control and Timeline chronic). MCR was determined according to definition. Multivariable logistic regression was used to examine the associations.

**Results:**

The overall prevalence of MCR was 11.5% (mean age = 71.62 ± 5.22). After adjusting for depression, anxiety, and cognitive function, positive control was associated with reduced risk of MCR (*OR* = 0.624, 95% *CI* 0.402–0.969, *P* = 0.036), subjective cognitive complaints (SCC) (*OR* = 0.687, 95% *CI* 0.492–0.959, *P* = 0.027), and gait speed (GS) (*OR* = 0.377, 95% *CI* 0.197–0.720, *P* = 0.003), respectively. Aging awareness was merely related to increased risk of MCR (*OR* = 1.386, 95% *CI* 1.062–1.810, *P* = 0.016).

**Conclusions:**

This study highlights the crucial associations of positive control and aging awareness with MCR and its components. Our results emphasize that positive belief in control and adaptive aging awareness might be promising targets for preventing MCR.

## Introduction

The aging population is increasing worldwide, and dementia poses a considerable challenge to all countries, including China, where individuals with dementia account for about 25% of the global dementia population [1]. Exploring pre-dementia syndrome provides an essential and feasible strategy for dementia prevention. Motoric cognitive risk syndrome (MCR) is a pre-dementia syndrome with the concurrence of subjective cognition complaints (SCC) along with slow gait speed (GS) in the absence of dementia and age-related mobility [2]. Given that MCR was considered as a strong predictor of cognitive impairment, and dementia [3, 4], it could be used as a powerful and easy tool for the early identification of individuals at risk of dementia in various clinical and community settings [5]. Furthermore, MCR increased the risk of falls and mortality [6, 7]. Thus, it is necessary to explore factors associated with MCR.

Several risk factors of MCR have been explored including older age, education, depression, and cardiovascular factors such as diabetes and stroke [7, 8]. However, only a few studies examined the psychological aspects of MCR. For example, higher neuroticism was linked to a higher risk of MCR [9], and openness was related to a lower risk of MCR [10]. Purpose in life and tangible social support were associated with lower MCR risks, respectively [11, 12]. Except for factors mentioned above, other psychosocial factors such as self-perceptions of aging (SPA) might be involved, for abundant evidence has been reported SPA was linked to components of MCR. Furthermore, exploring relations between SPA and MCR could potentially provide targets for MCR intervention, given SPA is malleable [13].

SPA could be explored from several perspectives, such as subjective age, views of aging, aging identities [14]. Evidence showed direct or indirect relationships between SPA, subjective cognitive complaints, and objective cognitive function. For example, negative views of aging were associated with subjective cognitive complaints in adults aged 50 or older [15], and the latter further aggravated negative thoughts of aging [16], forming a vicious cycle. Perceptions of aging modulated the relationship between frailty and cognitive decline, including global cognition, executive function, and attention [17].

Of note, more direct evidence about associations between SPA and MCR was from Stephan’ s study [18]. He found subjective age, a similar construct of SPA, increased about 60% likelihood of MCR and about 50% higher risk of incident MCR over a median follow-up 7.7 years. Since SPA is a relatively stable, and multiple-dimensional construct, subjective age fluctuated with situational context examined by a single question “Many people feel older or younger than they actually are” or “how old do individuals feel” [18, 19]. The associations between SPA and MCR was still unknown. Due to its excellent psychometric characteristics, multi-faced SPA could be measured using Brief version of Aging Perception Questionnaires (B-APQ) [20, 21]. Positive rather than negative control of SPA related to short delay memory, processing speed, and executive function in older adulthood [22]. Our previous study also found that older adults with lower positive control showed a cognitive decline [23]. Hence, we hypothesized that community-dwelling older adults with higher positive control over aging experience was associated with reduced risk of SCC and MCR.

Concerning the associations between SPA and gait speed, individuals feeling younger were associated with faster walking speed and reduced risk of walking speed decline, which is a vital predictor of mortality and cognitive decline [24]. Priming experiment also showed reinforcement of positive rather than negative views of aging significantly increased walking speed [25]. Thus, we hypothesized that community-dwelling older adults with positive aging experience reduce the risk of slow GS and MCR. The cross-sectional analysis found all five domains of SPA in APQ including aging awareness (Timeline Chronic dimension), and positive control were directly associated with walking speed after adjusting confounders. However, longitudinal analyses demonstrated that merely negative control and consequences domain rather than positive control over social relationships were related to changes in walking speed measured by Time Up and Go [26], rendering an elusive gap about the associations between positive or negative control with MCR. Evidence also showed that positive but not negative control exerted effects on functional disability, and depression which was also important influencing factors of MCR [27]. Thus, we hypothesized positive control was associated with MCR in the present study.

Findings showed that negative attitudes toward own aging in older adults predicted cognitive change over 20 years depending on depression [16]. Associations between anxio-depressive disorders, depression and MCR were found in large population-based cohort studies regardless of age group [28, 29]. Moreover, anxiety impacted processes of storage, retrieval, and visual recognition memory in older adults with subjective and cognitive complaints [30]. Thus, depression and anxiety were involved as psychological confounders.

This study examines the relationships between positive control, aging awareness, and MCR in Chinese community-dwelling older adults to provide interventional targets for community health promotors to prevent MCR.

## Methods

### Study population

In this cross-sectional study, all 1137 participants aged 65 years and older were recruited from a community health care center for routine physical examination in Xinxiang city of China from March 2021 to July 2021.100 participants were deleted due to more than 20% missing data in the survey, leading to a recruited rate of 91.9%. Trained interviewers collected data by face to face. The present study was approved by the Institutional Review Boards and Ethics Committee of Xinxiang Medical University (XYLL-2021004).

### Measurement

#### Measure of socio-demographic characteristics

Demographic factors included age, gender, marital status, education, living arrangement, monthly income, body mass index (BMI), lifestyle including diet, smoking, drinking history, and chronic illness, mainly including hypertension and diabetes mellitus. Living arrangement was examined by a question “Who do you usually live with?” and responses included living with family (sprouse, children and house maid) and living alone.

#### Definition of MCR

MCR was defined as a syndrome that combined slow gait speed (GS) and subjective cognitive complaints (SCC) without dementia and mobility disability following the criteria proposed by Verghese [2]. According to the criteria of MMSE, we excluded participants diagnosed with dementia. Mobility disability was excluded through self-reported difficulties in dressing, eating, walking, bathing, or showering, getting in or out of bed and using the toilet [31]. Subjective cognitive complaints were defined as self-reported or a third person reported cognitive disturbances, linked to feelings of persistent cognitive decline compared with a previously normal cognitive performance, without any deficits on objective testing [32]. The subjective cognitive complaints were assessed by asking “Do you have more difficulty to remember things?” Concerning slow gait speed, participants were asked to walk through a 4- meters long walking route in an open space. Slow gait speed was calculated by the average velocity of ≤ 1 SD below the age-and sex-specific population mean [4]. The following showed the cutoff value of slow GS: for female elderly aged ≤ 69, ≤ 0.78 m/s; female elderly aged 70–74, ≤ 0.72 m/s; female elderly aged ≥ 75, ≤ 0.61 m/s; For male elderly aged ≤ 69, ≤ 0.78 m/s; male elderly aged 70–74, ≤ 0.71 m/s; male elderly aged ≥ 75, ≤ 0.66 m/s.

#### Measurement of positive control and aging awareness in SPA

Positive control and aging awareness were assessed using two subscales of 17 items- B-APQ [21]. 3-itmes of Timeline Chronic domain represents constant aging awareness (I always classify myself as old; I am always aware of the fact that I am getting older; I feel my age in everything that I do), and another 3-items of Positive Control represents the belief that one can positively control over aspects of aging (The quality of my social life in later years depends on me; The quality of my relationships with others in later life depends on me; whether I continue to live life to the full depends on me); The Cronbach’s α coefficient of positive control, aging awareness in the study was 0.852, 0.719, respectively.

#### Assessment of depression and anxiety

Patient Health Questionnaire-9 (PHQ-9) was used to measure depressive symptoms over the last two weeks [23]. Higher scores indicate higher depressive symptoms. The Cronbach’s α coefficient in the study was 0.720. Anxiety symptoms were examined by Chinese version of Generalized Anxiety Disorder scale-7 item (GAD-7) over the past 2 weeks. Higher scores suggest higher anxiety symptoms. The Cronbach’s α coefficient in the study was 0.872.

#### Assessment of cognitive function

The Chinese version of Mini-Mental State Examination (MMSE) was used to evaluate global cognitive function with a total score of 30. Concerning the normal cognitive function, for participants with middle school and higher education, the score should exceed 24; for those with primary school education, the score should exceed 20, and for those illiteracy, the score was set at 17 [33]. Combined with MMSE and physical examination records, older adults with dementia were excluded. Normal cognitive function was also seen as an important covariable that should be adjusted in revealing the relationships between positive control, aging awareness, and MCR. The Cronbach’s α coefficient in the study was 0.612.

### Statistical analysis

SPSS 24.0 statistical software (IBM Corporation, Armonk, NY, USA) was used to perform statistical data. Data with non-normal distribution were expressed as mean rank. The Chi-square test and Mann–Whitney U test were used to compare between groups. Multivariable logistic regression analysis was used to estimate the odds ratio (OR) of association between positive control, aging awareness, and MCR and its components with 95% confidence interval (95% CI). The relatively healthy group was used as the reference group. Relatively healthy was defined here as the absence of MCR or subjective cognitive complaints or slow gait speed. The level of significance was set at 0.05.

## Results

### Characteristics of demographic variables and the prevalence of MCR

**Fig. 1:**
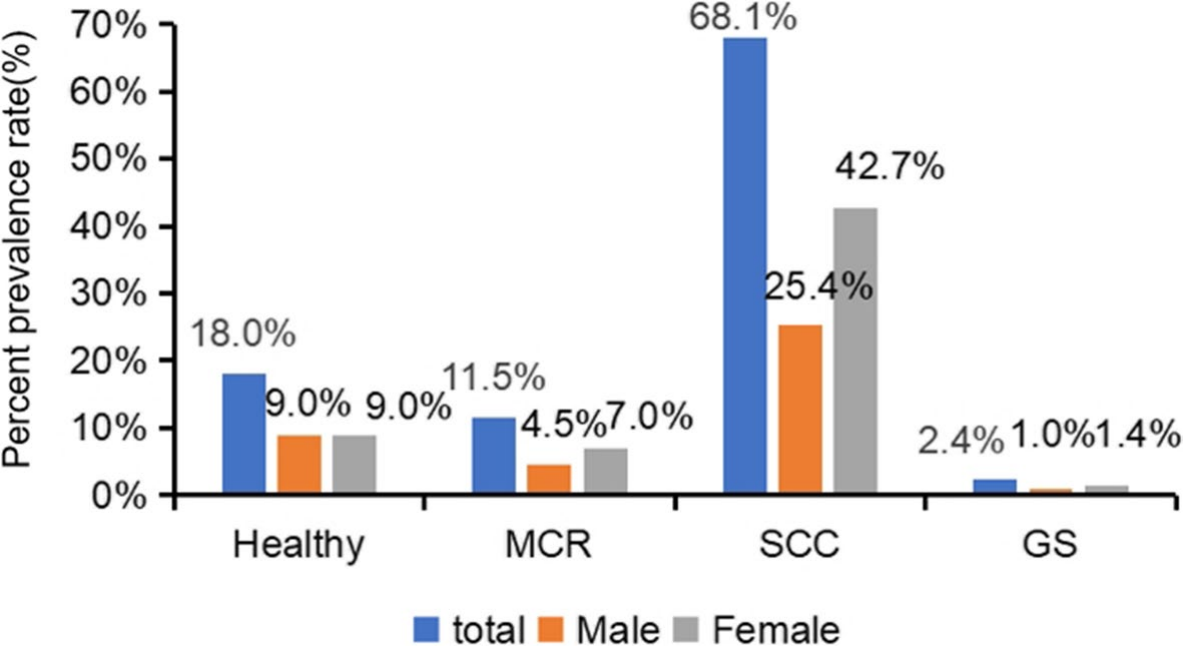
The prevalence of MCR and its components according to gender. MCR: motoric cognitive risk syndrome; SCC: subjective cognitive complaints; GS: gait speed

The mean age of participants was 71.62 ± 5.22 years. As shown in Fig. 1, of the 1137 community-dwelling older adults in the Central area of China, 131 (11.5%) participants had MCR; 774 (68.1%) participants had subjective cognitive complaints alone; 27 (2.4%) participants had slow gait speed alone, and 205 maintained relatively healthy (18%). As shown in Table 1, more females (*n* = 684, 60.2%) than males (*n* = 453, 39.8%) participated in this study, but no significant difference (50.2% and 49.8%, respectively) was observed in the relatively healthy group. As shown in Fig. 1 and Table 1, in the MCR group (11.5%, totally) and SCC group (68.1%), there were more female elderly (7%, and 42.7%, respectively). Compared with older adults in the relatively healthy group, older adults with MCR were more widows or divorced, had a lower educational level, had a vegetarian diet and were more likely to have a drinking history(*P* ≤ 0.05).

**Table 1:** Participants’ characteristics in Healthy, MCR, SCC, and GS group in Chinese community-dwelling adults (*n* = 1137)

Characteristics	Relatively Healthy n (%)	MCR n (%)	Only SCC n (%)	Only GS n (%)	*χ*^2^	*P value*
	205 (18)	131 (11.5)	774 (68.7)	27 (2.4)		
Gender						
Men	102 (49.8)	51 (38.9)	289 (37.3)	11 (40.7)	10.485	0.015
Female	103 (50.2)	80 (61.1)	485 (62.7)	16 (59.3)		
Marital status						
Married	181 (88.3)	102 (77.9)	647 (83.6)	26 (96.3)	9.650	0.022
Widowed or divorced	24 (11.7)	29 (22.1)	127 (16.4)	1 (3.7)		
Education						
Illiterate	8 (3.9)	17 (13.0)	46 (6.0)	3 (11.2)	35.426	<0.001
Primary	29 (14.1)	30 (22.9)	101 (13.0)	4 (14.8)		
Middle school	66 (32.2)	38 (29)	202 (26.1)	12 (44.4)		
Tertiary or above	102 (49.8)	46 (35.1)	424 (54.9)	8 (29.6)		
Monthly income (¥)						
< 1000	15 (7.3)	16 (12.2)	72 (9.3)	3 (11.1)	15.824	0.071
1000–2999	64 (31.2)	47 (35.9)	207 (26.7)	7 (25.9)		
3000–4999	87 (42.4)	56 (42.7)	332 (42.9)	14 (51.9)		
≥ 5000	39 (19.1)	12 (9.2)	163 (21.1)	3 (11.1)		
Living arrangement						
Living with family	191 (93.2)	126 (96.2)	718 (93.5)	27 (100)	3.362	0.339
Living alone	14 (6.8)	5 (3.8)	50 (6.5)	0 (0)		
Hypertension						
yes	127 (62)	70 (53.4)	456 (59)	14 (51.9)	2.941	0.401
no	78 (38)	61 (46.6)	317 (41)	13 (48.1)		
Diabetes						
yes	178 (86.8)	110 (84.0)	670 (86.7)	25 (92.6)	1.618	0.655
no	27 (13.2)	21 (16.0)	103 (13.3)	2 (7.4)		
Diet						
Balanced	125 (61.3)	50 (38.2)	379 (49)	11 (40.7)	23.220	0.001
Carnivorous	8 (3.9)	4 (3)	36 (4.7)	3 (11.2)		
Vegetarian	71 (34.8)	77 (58.8)	358 (46.3)	13 (48.1)		
Smoking						
yes	147 (71.7)	99 (75.6)	606 (78.4)	19 (70.4)	4.797	0.187
no	58 (28.3)	32 (24.4)	167 (21.6)	8 (29.6)		
Drinking history						
yes	138 (67.3)	100 (76.9)	579 (74.9)	16 (59.3)	8.369	0.039
no	67 (32.7)	30 (23.1)	194 (25.1)	11 (40.7)		

### Comparison of related variables between different groups

Table 2 shows participants in the MCR group were the oldest (73.19 ± 6.42 years), had the highest level of depressive and anxiety symptoms (mean rank = 646.22, 619.78, respectively), and had the lowest level of cognitive function (mean rank = 439.69) among groups. Older adults in the MCR group had the highest level of aging awareness (3.51 ± 0.80) years and a moderate level of positive control (mean rank = 534.61). In contrast, the relatively healthy group and GS group had the highest and lowest level of positive control (mean rank = 606.08, 481.70, respectively).

**Table 2:** Characteristics and comparing among groups with healthy, MCR, SS, and GS (*n* = 1137)

Characteristics (Mean ± SD or Mean rank)	Relatively Healthy (*n* = 205)	MCR (*n* = 131)	Only SCC (*n* = 774)	Only GS (*n* = 27)	*F/χ*^*2*^	*P value*
Age	71.35±4.63	73.19±6.42	71.44±5.07	71.48±6.13	4.502	0.004
BMI	24.62±3.09	25.11±3.27	24.63±3.03	25.17±2.49	1.164	0.322
Depression	503.75	646.22	574.77	524.37	18.680	<0.001
Anxiety	503.86	619.78	576.94	568.94	16.206	0.001
MMSE	606.12	439.69	582.55	526.15	25.294	<0.001
Aging awareness	3.04±1.09	3.51±0.80	3.17±0.90	3.14±1.09	6.818	<0.001
Positive control	606.08	534.61	568.05	481.70	9.420	0.024

Note: *BMI* body mass index

As shown in Table 3, the relatively healthy group served as the reference group and the multivariable logistic regression results in Model 3 showed that aging awareness and depression were associated with increased risk of MCR (*OR* = 1.386, 95% *CI* 1.062-1.810, *P* = 0.016; *OR* = 1.195, 95% *CI* 1.045-1.368, *P* = 0.009, respectively). Cognitive function was associated with reduced risk of MCR (*OR* = 0.872, 95% *CI* 0.775-0.981,* P* = 0.022). Similarly to results in crude model 1 and adjusted model 2, positive control was significantly associated with decreased risk of MCR, SCC and GS, respectively (*OR* = 0.624, 95% *CI* 0.402-0.969, *P* = 0.036; *OR* = 0.687, 95% *CI* 0.492-0.959, *P* = 0.027; *OR* = 0.377, 95% *CI* 0.197-0.720, *P* = 0.003, respectively) in model 3. Positive control was found an important protective factor after adjusting for significant variables examined in univariable analysis such as education, marital status, diet, depression, cognitive function, indicating that positive control might become an important target for preventive intervention of both SCC, GS, and MCR. Aging awareness was only related to increased risk of MCR but not its components.

**Table 3:** Multivariable analysis of the association of positive control, aging awareness in SPA with MCR, SCC, and GS (*n* = 1137)

	MCR			Only SCC			Only GS		
	*OR*	*95%CI*	*P value*	*OR*	*95%CI*	*P value*	*OR*	*95%CI*	*P value*
Model 1									
Aging awareness	1.705	1.335–2.179	< 0.001	1.147	0.974–1.349	0.099	1.058	0.687–1.629	0.797
Positive control	0.606	0.398–0.921	0.019	0.716	0.516–0.993	0.045	0.406	0.218–0.758	0.005
Model 2									
Aging awareness	1.588	1.226–2.056	< 0.001	1.143	0.965–1.354	0.122	1.058	0.685–1.634	0.798
Positive control	0.594	0.385–0.916	0.018	0.681	0.490–0.945	0.022	0.378	0.199–0.717	0.003
Model 3									
Age (year)	1.023	0.977–1.072	0.329	0.998	0.965–1.032	0.887	1.032	0.939–1.114	0.602
Male	1.141	0.595–2.184	0.692	0.657	0.425–1.017	0.060	0.251	0.059–1.065	0.061
Education									
Illiterate	2.450	0.782–7.676	0.124	1.032	0.411–2.593	0.946	2.983	0.478–18.628	0.242
Primary	1.340	0.650–2.762	0.428	0.612	0.363–1.032	0.066	1.147	0.285–4.621	0.847
Middle school	1.311	0.752–2.288	0.340	0.670	0.465–0.965	0.032	1.942	0.737–5.118	0.179
Married	0.691	0.350–1.366	0.287	0.782	0.471–1.298	0.342	4.809	0.586–39.47	0.144
Diet									
Balanced diet	0.469	0.287–0.766	0.002	0.677	0.481–0.952	0.025	0.529	0.216–1.297	0.164
Meat diet	0.495	0.137–1.784	0.282	0.962	0.420–2.206	0.928	1.626	0.352–7.514	0.534
Drinking history (No)	1.258	0.636–2.487	0.510	1.007	0.643–1.75	0.976	0.237	0.057–0.989	0.048
Aging awareness	1.386	1.062–1.810	0.016	1.071	0.899–1.276	0.440	1.042	0.667–1.630	0.856
Positive control	0.624	0.402–0.969	0.036	0.687	0.492–0.959	0.027	0.377	0.197–0.720	0.003
Depression	1.195	1.045–1.368	0.009	1.098	0.982–1.227	0.101	0.967	0.720–1.297	0.821
MMSE	0.872	0.775–0.981	0.022	0.964	0.884–1.051	0.409	0.911	0.742–1.119	0.376
Anxiety	1.093	0.959–1.245	0.183	1.097	0.990–1.217	0.078	1.078	0.843–1.378	0.548

Notes: Model 1: crude model; Model 2: adjusted for age, gender, education, married status, diet, drinking history. Model 3: ajusted for Model 2 plus depression, MMSE and anxiety

Additionally, compared with a vegetarian diet, a balanced diet was associated with reduced risk of MCR (*OR* = 0.469, 95% *CI* 0.287-0.766,* P* = 0.002) and SCC (*OR* = 0.677, 95% *CI* 0.481-0.952,* P* = 0.025). Individuals with a middle school education and above was related to reduced risk of SCC (*OR* = 0.670, 95% *CI* 0.465-0.965,* P* = 0.032). Interestingly, non drinking history was linked to a reduced risk of GS (*OR* = 0.273, 95% *CI* 0.057-0.989,* P* = 0.048).

## Discussion

### Prevalence of MCR and related factors

The present study extended previous studies by providing the prevalence of MCR among community-dwelling older adults in the Central area of China. The prevalence of MCR was 11.5% in the present study (mean age = 71.62 ± 5.22), which was among the reported range of 9.6% ~ 12.7%(9.6% in Beijing, China [31], 10% worldwide [5], 12.7% in East China, 10.7% in West China [8], and 10.4% in a nationally Chinese sample [34]. Slightly different results might be related to assessment tools, participants of diverse ages, ethics, and senior hospital departments [5, 8, 31, 34]. Our results found aging awareness, and positive control were associated with MCR risk after adjusting for related confounders.

In the present study, aging awareness was only related to increased risk of MCR but not its components. This might be related to different measures of SPA. Althgouh both views of aging and subjective age could reflect SPA to some degree, there were some differences regarding asscociations with SCC. View of aging means attitudes towards both psychological loss, physical decline and psyhcological growth according to Attitudes to Aging questionnaire. Aging aware in B-APQ reflects awareness of one’s aging or age, more similar to subjective age. View of aging contributed to all dimensions of cognitive complaints such as abilities to remember, recognize, recall or understand, et al. [15]. However, subjective age was not associated with complaints about memory [15]. SCC in this study are memory-specific cognitive complaints and SPA is a multiple domian construct. Given positive control was associated with MCR, SCC and gait speed, our finding indicates the relationship between SPA and SCC might be domain-specific and warrant further studies.

Our result that positive control was associated with the reduced risk of MCR was easy to be understood for previous evidence linked positive control to MCR components in older adults. Older adults with higher control beliefs had better executive functioning than counterparts [35]. Similarly, high levels of personal mastery predicated fewer subjective memory complaints [36]. Although only negative control rather than other dimensions of SPA predicted lower walking speed [26], older adults who endorsed more positive SPA were more likely to exhibit a stable, fast gait at maximum speed [37].

Several mechanisms were likely to be involved in the association between positive control and the lower risk of MCR. First, positive control over aging differs from negative control focusing on physical autonomy. Positive control in SPA refers to adaptive coping, mainly emphasizing positive beliefs about social relationships. Quality rather than quantity of social relationships benefited to older adults’ global cognitive function [38]. Social activity or a more extensive social network was positively linked to faster gait speed and physical fitness [39, 40]. Conversely, social isolation was an independent risk of further physical performance decline detected by the Timed Up and Go (TUG) test [41]. Perceived social control mediated the associated social support and physical function and depressive symptoms, which were related to MCR found in our study and previous studies [42, 43]. In line with previous studies [34, 44], depression was associated with MCR and risk factors for a transition of MCR to dementia.

Second, positive control also includes beliefs to live life to the full (whether I continue to live life to the full depends on me). Meaning-in-life, including interpersonal relationship, was associated with subjective memory complaints [45]. Compared with other psychological factors of MCR such as personality, and purpose in life, positive control in SPA based on self-regulatory framework were more modifiable [13, 21].

Of note, compared to participants with a vegetarian diet, older adults with a balanced diet had lower risk of MCR. Our results were different from one study that certain amount of cholesterol was associated with lower risk of MCR in West China [8]. The reasons might be related to metabolism, as an umbrella review demonstrated that vegetarian diets benefit reduced risk of chronic diseases such as stroke, cancer risk which is not found risk factors of MCR [7], but a vegetarian diet might do harm to one-carbon metabolism markers by reducing the concentrations of Vitamin B12, and increasing concentrations of homocysteine [46]. An international consensus statement showed that elevated plasma homocysteine was a risk factor for the development of cognitive decline, and dementia in older adults [47]. The effect of Vitamin B12 supplementation has not been demonstrated effective [48]. Homocysteine rather than Vitamin B12 was associated with gait speed decline in healthy older adults [49]. Thus, homocysteine levels might serve as a mechanism associated with the risk of MCR with a vegetarian diet, which warrants future studies.

It is much better to keep thin in Chinese traditional culture, just as a Chinese proverb says, “money cannot make one thin in his old age.” Maladaptive aging beliefs might promote older adults to maintain a vegetarian diet. A study found positive SPA (capturing aging knowledge and control) promoted healthy eating patterns [50]. Older adults with a balanced diet, adaptive aging awareness, and positive control about aging might confer role models for preventative interventions of MCR in community settings. Consistent with previous studies [51], a drinking history of older adults was merely associated with reduced risk of gait speed compared with non drinkers. This phenomenon was likely to be related to different levels of alcohol use [51]. The relationships of drinking and MCR need further examination [8].

Although a home-based exercise program with telephonic coaching were potentially safe and feasible in preventing adverse outcomes in older adults with MCR [52]. Double-task Happy exercises incorporated whole-body movement, and cognitive activities might improve cognitive and increase robustness among older adults with MCR [53]. Interventions to prevent MCR and MCR transitions to dementia are still few. Our findings have several implications. First, interventions to reduce the risk of MCR could add strategies with positive aging awareness and control over social relationships, such as cognitive behavioral therapy, savoring life lessons, and physical exercise [54, 55]. Second, educational programs should renew older adults’ awareness about aging and a balanced diet to prevent MCR.

### Limitations and future work

This study is the first to identify positive control and aging awareness associations with pre-dementia syndrome MCR. There are also some limitations. First, a cross-sectional study limited the exploration of the casual relationship of positive control and aging awareness of SPA with MCR. Our cohort study is ongoing and will provide more information. Second, we didn’t explore the potential mechanisms behind the relations. It warrants further studies given SPA conferred physical and psychological effects through behavioral, lifestyle, and psychological mechanisms [23]. Third, there were still other confounders involved.

## Conclusions

This study provided the prevalence of MCR among community-dwelling older adults in the Central area of China. Future MCR preventive interventions should consider positive control and aging awareness of older adults.

## Data Availability

The datasets used and/or analyzed during the current study are available from the corresponding author on reasonable request.

## Abbreviations

MCR: Motoric cognitive risk syndrome
SPA: Self-perceptions of aging
SCC: Subjective cognitive complaints
GS: Gait speed
B-APQ: Brief version of Aging Perception Questionnaires
PHQ-9: Patient Health Questionnaire-9
MMSE: Mini-Mental State Examination
OR: Odds ratio
CI: Confidence interval
TUG: Timed Up and Go
